# Giant circulating malignant cells spotted in feathered edge of blood film

**DOI:** 10.1002/jha2.487

**Published:** 2022-06-13

**Authors:** Robert J. Guo, Tyler W. Smith

**Affiliations:** ^1^ Department of Pathology and Laboratory Medicine University of British Columbia Vancouver British Columbia Canada; ^2^ Department of Pathology and Laboratory Medicine Vancouver General Hospital Vancouver British Columbia Canada

1

A 20‐year‐old male presented with a 4‐week history of dry cough, night sweats, weight loss, and fever. Blood tests showed normocytic anemia (hemoglobin 71 g/L) and severe thrombocytopenia (platelets 17 × 10^9^/L). His blood film demonstrated leukoerythroblastosis, occasional red blood cell fragments, and giant cells in the feathered edge (Figure 1, panel A; Wright–Giemsa stain, original magnification ×400). Imaging revealed a large anterior mediastinal mass. Bone marrow biopsy revealed a hypercellular marrow with extensive involvement by high‐grade malignant cells (Figure 1, panel B—aspirate; Wright–Giemsa stain, original magnification ×400; panel C—trephine biopsy; hematoxylin and eosin stain, original magnification ×200). Immunohistochemical staining only revealed CD43^+^, pankeratin^+^, and weak CD30 (Figure 1, panels D–F).

**FIGURE 1:**
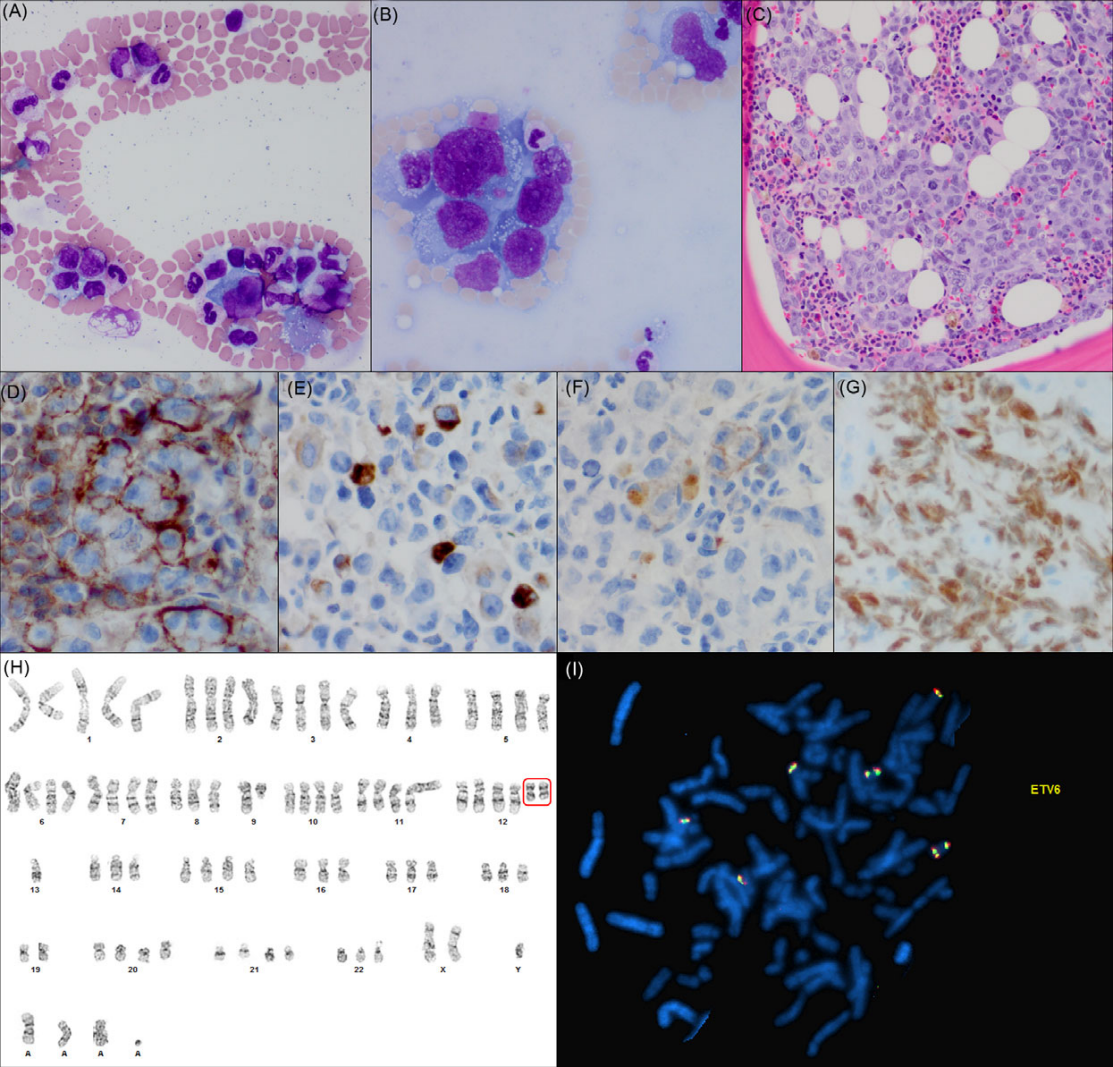
Large nonhematopoietic cells observed in the feathered edge of blood film (panel A), bone marrow aspirate (panel B), and bone marrow biopsy (panel C). The cells were CD43, pankeratin, weak CD30, and Sallike protein 4 (SALL4) (panels D–G). The final diagnosis was nonseminomatous germ cell tumor, not further classified. This was further supported by the presence of two isochromosome 12p, identified on karyotype and metaphase Fluorescence In Situ Hybridization (FISH) with the translocation‐Ets‐leukemia virus‐6 (ETV6, 12p13.2) probe (panels H–I).

The mediastinal mass was biopsied and showed similar results, as well as positivity for Sal‐like protein 4 (SALL4; Figure 1, panel G) and epithelial membrane antigen (EMA). Serum alphafetoprotein was also elevated. Karyotype and metaphase Fluorescence In Situ Hybridization (FISH) from the bone marrow showed two isochromosome 12p (Figure 1, panels H–I). The final diagnosis was nonseminomatous germ cell tumor, not further classified.

This case illustrates the importance of scanning the feathered edge of blood films to check for circulating metastatic cells. While often discussed in theory and seldom seen in practice, it is rare cases like this that reaffirm the importance of checking the feathered edge.

## CONFLICT OF INTEREST

The authors declare no conflict of interest.

## AUTHOR CONTRIBUTIONS

Robert Guo and Tyler Smith helped sign out the hematopathology report on the bone marrow specimen for this case, as well as in writing and editing this manuscript.

